# Prophylactic Heme Arginate Infusion for Acute Intermittent Porphyria

**DOI:** 10.3389/fphar.2021.712305

**Published:** 2021-10-06

**Authors:** Hung-Chou Kuo, Chia-Ni Lin, Yi-Fen Tang

**Affiliations:** ^1^ Department of Neurology, Chang Gung Memorial Hospital-Linkou Medical Center, Chang Gung Memorial Hospital and College of Medicine, Taoyuan, Taiwan; ^2^ Department of Laboratory Medicine, Chang Gung Memorial Hospital, Taoyuan, Taiwan; ^3^ Department of Medical Biotechnology and Laboratory Science, College of Medicine, Chang Gung University, Taoyuan, Taiwan; ^4^ Department of Pharmacy, Linkou Chang Gung Memorial Hospital, Taoyuan, Taiwan

**Keywords:** menopause, porphyric attack, annual attack rate, heme arginate, heme prophylaxis, acute intermittent porphyria

## Abstract

**Objectives:** This study aimed to evaluate the efficacy of long-term weekly prophylactic heme arginate (HA) infusions in reducing attack frequency and severity in female AIP patients.

**Methods:** We report the results of five female AIP patients with frequent recurrent attacks (>9/year) before and after institution of weekly prophylaxis with heme arginate (3 mg/kg body weight). All five cases had confirmed disease-associated mutations in the porphobilinogen deaminase gene, and all had received genetic and clinical counseling about AIP.

**Results:** In the five included patients, average annual attack rate (AAR) in the year prior to HA prophylaxis was 11.82 (range 9.03–17.06), and average total HA usage was 32.60 doses (range: 13.71–53.13). After 2.58–14.64 years of HA prophylaxis, average AAR was reduced to 2.23 (range 0.00–5.58), and attack severity (i.e., doses required per attack) was reduced from 2.81 to 1.39 doses/attack. Liver and renal function remained stable during weekly administration of HA prophylaxis. The most common complications were port-A catheter-related events. No other complications or safety concerns occurred with long-term use of HA prophylaxis.

**Conclusion:** Our study demonstrated women with AIP receiving weekly prophylactic HA infusions resulted in fewer episodes that required acute HA treatment while maintaining stable renal and liver function. Weekly prophylactic HA infusions effectively prevent frequent porphyric attacks and reduce attack severity.

## Introduction

Acute intermittent porphyria (AIP) is an autosomal hereditary disease caused by dominant negative mutation in the porphobilinogen deaminase (PBGD) gene and resulting accumulation of potentially neurotoxic porphyrin precursors of heme biosynthesis. AIP patients experience periodic severe abdominal pain and sympathetic nervous system over-activity (e.g., hypertension, palpitation, tachycardia), often requiring emergency hospitalization. Other neurological symptoms may include delirium, seizures, motor paresis, and hysteria, often leading to a neuropsychiatric misdiagnosis and subsequent mistreatment (Jain et al., 2011). AIP attacks are more commonly observed in females after puberty, and sex hormones are considered to be a precipitating factor in disease onset. Cyclic acute AIP attacks in females are closely associated with the luteal phase of the menstrual cycle (Andersson et al., 2003; Pischik and Kauppinen, 2006). In some female patients, acute flares are predictable and recur regularly 7–10 days prior to menses every one or 2 months. Other triggers of AIP clinical symptoms include infection, fasting, and medications, although activating factors often remain unidentified (Siegesmund et al., 2010).

Treatment of acute AIP attacks involves symptomatic treatment and suppression of hepatic rate-limiting enzymes of heme biosynthesis (i.e., 5-aminolevulinic acid synthase 1, or ALAS-1). Glucose, which inhibits ALAS1 by affecting peroxisome proliferator-activated receptor gamma coactivator1-alpha (Handschin et al., 2005), could be considered in situation of mild attacks. For severe acute attacks, intravenous heme is the most effective therapy as it provides exogenous heme and down-regulates ALAS1 transcription which in turn, results in a rapid reduction in the overproduction of ALA and PBG. Typically, acute porphyric attacks last no longer than 1–2 weeks and can be treated efficiently with 3–5 days of intravenous heme therapy. In Taiwan, intravenous heme therapy is only available in the form of heme arginate (HA), while hematin is available in the United States. HA has been recommended as the initial treatment for acute porphyric attacks because of its increased stability and safety over hematin (Mustajoki and Nordmann, 1993; Anderson, 2019).

Severe AIP attacks can be frequent, potentially life-threatening, and lead to chronic deterioration in neurological, liver and kidney function, reducing patients’ quality of life as well as increasing financial burden (Neeleman et al., 2018). Recently, an exciting RNA interference (RNAi) therapy, givosiran, was FDA-approved for reducing the severity and frequency of porphyric attacks in acute hepatic porphyria (Balwani et al., 2020; Honor et al., 2021; Thapar et al., 2021). but its accessibility and pricing may remain limiting issues for use (Massachi et al., 2020; Thapar et al., 2021). Other prevention strategies for AIP attacks include hormone-suppression therapy (Anderson et al., 1990; Herrick et al., 1990; Kauppinen and Mustajoki, 1992), prophylactic heme therapy (Kauppinen, 2005; Yarra et al., 2019), and in extreme cases, liver transplantation (Soonawalla et al., 2004; Dowman et al., 2012; Balwani et al., 2017; Stein et al., 2017). Prophylactic administration of human heme could also be considered for childbearing age woman who experienced an acute porphyria flare during her first pregnancy and wish to conceive for second pregnancy (Vassiliou et al., 2020).

Recurrent severe attacks affect about 3–8% of patients with acute porphyria (Schmitt et al., 2018), however, reports on the use of prophylactic HA therapy were limited. In our medical center, prophylactic HA therapy has been offered to carefully selected AIP patients, approximately 5% of AIP patients are receiving scheduled prophylactic HA infusion. This study aimed to demonstrate the safety and efficacy of long-term weekly prophylactic HA infusions in preventing frequent porphyric attacks in patients with AIP.

## Materials and Methods

### Study Design

Retrospective review of medical records was conducted. The hospital historical records that were evaluated was from the first available record of each case patient to July 2020. Demographics, treatment history (since their initial presentation to the hospital), and clinical outcomes of interest were extracted from patients’ medical records in the emergency room, inpatient ward, and outpatient clinic.

### Study Subjects

Female patients receiving weekly prophylactic HA (Normosang^®^, Orphan Europe) infusions at Chang Gung Memorial Hospital (CGMH), Linkou Medical Center, Taiwan, and who met the following criteria were included: 1) confirmed mutation in the PBGD gene and diagnosed with AIP; 2) Patients with frequent porphyric attacks, defined as having at least 9 attacks in the year prior and receiving more than one HA infusion for the attacks (The data for ALA and PBG level associated with reported attacks is presented in Supplementary Table S1); 3) received genetic counseling and were educated to avoid potential triggering factors of AIP acute attacks; and 4) had been receiving weekly infusions of HA (3 mg/kg body weight) prophylactically to prevent AIP attacks. Electrophysiological findings of the patients with neurologic porphyria have been reported in our prior studies (Kuo et al., 2011; Wu et al., 2015; Kuo et al., 2016; Lin et al., 2018).

### Ethical Considerations

The study protocol was approved by the institutional review board of CGMH (202000914B0), and the study was conducted in accordance with the Declaration of Helsinki. Written informed consent of included patients was waived by the same committee because of the retrospective nature of the study, data analysis were performed using only the de-identified data.

### Main Outcome Measures

The main efficacy outcomes include the frequency and the severity of porphyric attacks. An acute attack is defined as an attack requiring hospitalization, urgent healthcare visits, and treatment with at least one heme arginate infusion. The frequency of attacks is defined as the annual rate of attacks (annualized attack rate, or AAR). Attack severity is represented by the number of heme infusions (equivalent to days of receiving heme therapy) during an attack, with HA administered at 3 mg/kg body weight/day. Safety evaluation included estimated glomerular filtration rate (eGFR), serum aspartate transaminase (AST) levels, serum alanine transaminase (ALT) levels, and renal and liver ultrasound findings. Thrombophlebitis, port A infection and replacement, and transferrin saturation index (% of serum iron related to TIBC) are included as complications associated with HA infusions.

### Statistical Analysis

Patients’ demographic and clinical characteristics for the period before HA treatment were summarized for each patient. Changes in attack severity after initiating HA treatment, including number of attacks and AAR, total doses of HA required for attacks, and duration of treatment was also summarized for each patient. Treatment duration in years is represented as the total number of treatment days/365.25. Personal dose usage per year was calculated as total doses of HA used divided by duration, and AAR was calculated as total number of porphyric attacks divided by duration. Averages were calculated for the number of porphyric attacks, total doses of HA required for porphyric attacks, duration of treatment period, doses per year, and overall AAR for all five patients. All data were arranged and graphed using Microsoft Excel (Microsoft, Redmond, WA, United States).

Role of the funding source: The funding agency had no role in study design, data collection and data analysis, decision to publish, or preparation of the manuscript.

## Results

A total of five female patients who met the study criteria were included. Patient demographics, clinical characteristics and brief medical history are summarized in Table 1. ALA and PBG data for all 5 patients are summarized in Supplementary Table S1, biochemical elevation of ALA and PBG are evident. AARs for each patient individually and averages for overall patients are listed in Table 2. Average AARs decreased from 11.82 at 1 year prior to prophylaxis to 2.25 during/after prophylactic HA treatment. Reduced attack rates were sustained throughout the follow-up period (average 8.57 years) with continued use of weekly prophylactic HA infusions.

**TABLE 1:** Selected demographic and clinical characteristics of AIP patients for period before heme arginate prophylaxis.

Case No.	#1	#2	#3	#4	#5
Sex	F	F	F	F	F
Current Age (years)	51–55	51–55	21–25	31–35	51–55
Age of onset (years)	36	33	16	20	30
PBGD gene mutation	c.652G > A exon 11	c.77G > A exon 2	c.652G > A exon 11	c.33+5G > A at IVS1	c.973C> T exon 14
Predicted change in protein	p. Gly218Arg	p. Arg26His	p. Gly218Arg	Splicing aberration	p. Arg325X
Neurological manifestations except for visceral symptoms before diagnosis, CNS	None	neuropsychologic symptoms	None	Convulsion and resulted in rhabdomyolysis	Conscious impairment
Neurological manifestations except for visceral symptoms before diagnosis, PNS	None	Sensorimotor polyneuropathy	None	None	Motor paresis, severe neuralgia, dysarthria
Clinical characteristics					
Severity of disease ((1-year prior to prophylaxis)	9 attacks, total of 25 hemin doses	13 attacks, total of 56 hemin doses	17 attacks, total of 30 hemin doses	10 attacks, total of 33 hemin doses	10 attacks, total of 19 hemin doses
eGFR prior to prophylaxis, (ml/min/1.73m^2^)	65.6	24	144	74.4	16
ALT prior to prophylaxis, (U/L)	27	18	20	24	27
AST prior to prophylaxis, (U/L)	27	25	20	33	28
Years of receiving weekly prophylaxis (Year of initiation)	14.64 (2005 Nov)	7.34 (2013 Feb)	4.36 (2014 Jan–2018 Jun^a^)	13.91 (2006 Aug)	2.58 (2017 Nov)

a Case #3 was recruited to a phase 3 trial of RNAi therapeutic givosiran for acute intermittent porphyria (ENVISION trial), discontinued heme arginate prophylactic treatment during 2018 June. AIP, acute intermittent porphyria; CNS, central nervous system; PNS, peripheral nervous system.

**TABLE 2:** Summary of changes in attack severity after initiating heme arginate prophylactic treatment.

	HA for acute attack 1-year before prophylactic treatment				HA prophylactic treatment from initiation to menopause					HA prophylactic treatment from menopause to present (2020 June)					HA prophylactic treatment from initiation to present (2020 June)				
Case #	Attacks (n)	HA use (dose)	Doses/year	AAR^b^	Duration^a^ (years)	Attacks (*n*)	HA use (dose)	Doses/year	AAR^b^	Duration^a^ (years)	Attacks (n)	HA use (dose)	Doses/year	AAR^b^	Duration^a^ (years)	Attacks (n)	HA use (dose)	Doses/year	AAR^b^
#1	9	25	13.71	9.03	11.64	26	40	3.44	2.23	3.00	5	2	0.67	1.67	14.64	31	42	2.87	2.12
#2	13	56	53.13	13.01	5.59	40	119	21.27	7.15	1.75	1	3	1.71	0.57	7.34	41	122	16.61	5.58
#3	17	30	25.25	17.06	—	—	—	—	—	—	—	—	—	—	4.36^d^	7^d^	9^d^	2.07^d^	1.61^d^
#4	10	33	33.11	10.03	—	—	—	—	—	—	—	—	—	—	13.91	27	36	2.59	1.94
#5	10	19	19.12	10.06	—	—	—	—	—	3.58^c^	10^c^	19^c^	5.30^c^	2.79^c^	2.58	0	0	0.00	0.00
Average	11.8	32.6	32.60	11.82	9.22	33.0	79.5	12.36	4.7	2.78	5.3	8.0	2.56	1.7	8.57	21.2	41.8	4.83	2.25

a Duration, total number of days receiving treatment/365.25.

b AAR, annualized attack rate (=total number of porphyria attacks/duration).

c For case #5, duration, attacks, and doses of heme arginate given for porphyric attacks after menopause to starting observation 1-year for frequent and severe attack were not included.

d For Case #3, duration was set as date of starting heme arginate prophylactic treatment until date of screening for the clinical trial (2018/Jun./8).

### Time Course Description of Treatments Prior to Prophylaxis

**FIGURE 1:**
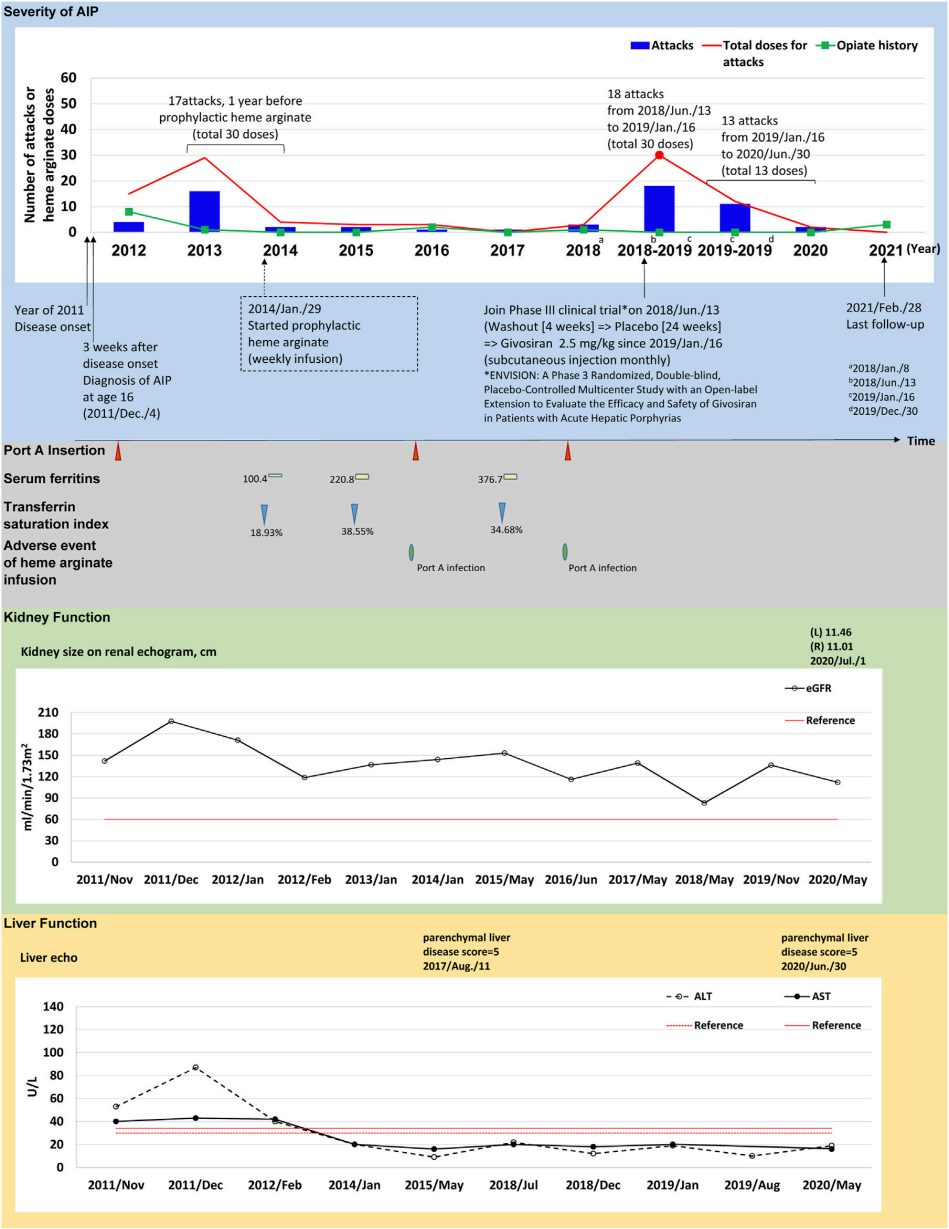
The tracked information of AIP severity, adverse events, kidney and liver function for patient #3. AIP severity is identified by numbers of attacks and total heme arginate doses for porphyric attacks treated. Black dots indicate the most severe attack in the past, which was treated using heme arginate. Red dots indicate AIP severity status within 1-year before receiving prophylactic heme arginate. A dotted frame marks the initiation date of initial prophylactic heme arginate infusion. Port A insertion and adverse events of heme arginate infusion are shown as red triangle with given examined date, Port A-related infection is shown as a green oval. Serum ferritin is indicated as yellow square. Transferrin saturation index (% of serum iron relative to TIBC) is shown as blue triangle. Kidney function is recorded as eGFR level, and sizes of left (L) and right (R) side of kidney along with examined date. Liver function is recorded as ALT and AST levels, and parenchymal liver disease score along with examined date.

**FIGURE 2:**
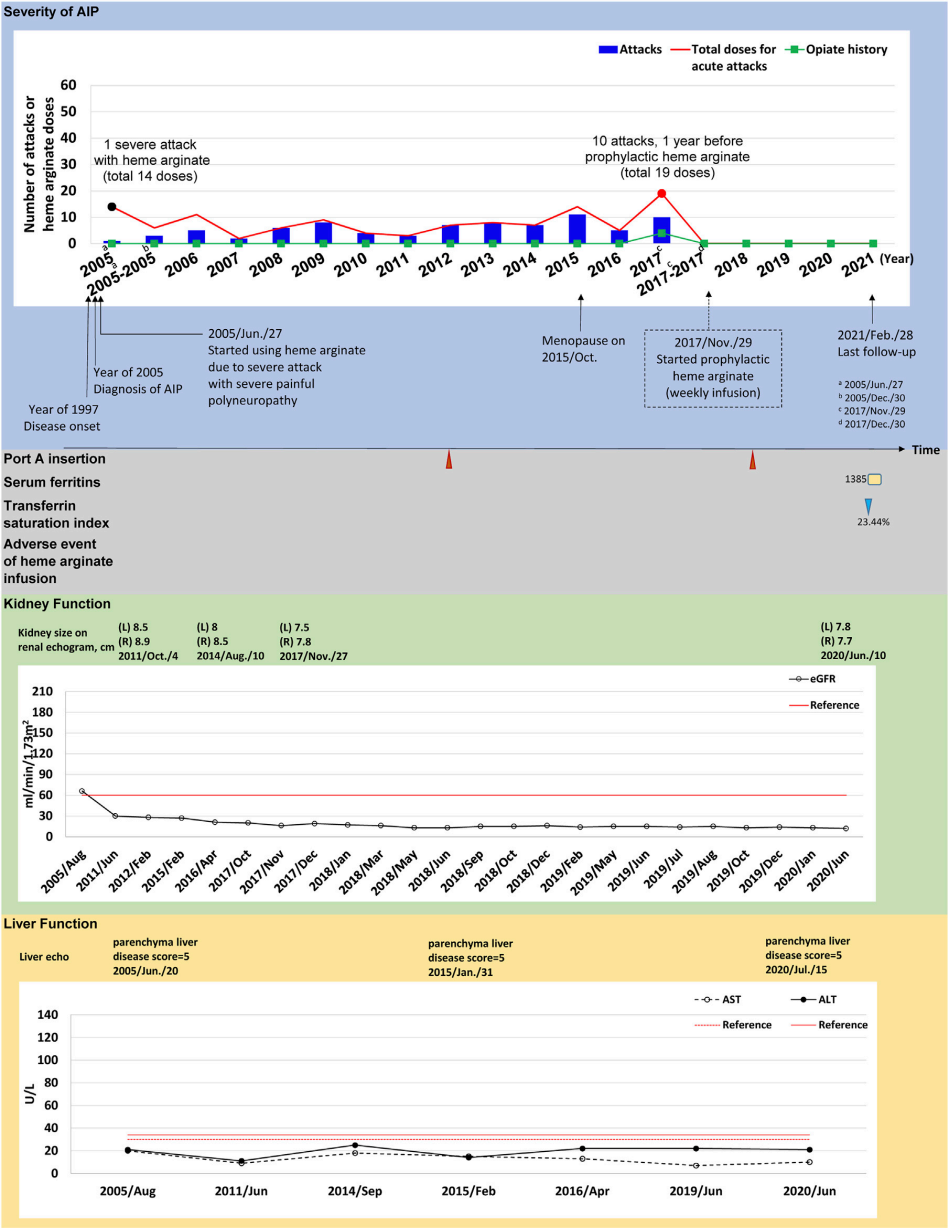
The tracked information of AIP severity, adverse events, kidney and liver function for patient #5. AIP severity is identified by numbers of attacks and total heme arginate doses for porphyric attacks treated. Black dots indicate the most severe attack in the past, which was treated using heme arginate. Red dots indicate AIP severity status within 1-year before receiving prophylactic heme arginate. A dotted frame marks the initiation date of initial prophylactic heme arginate infusion. Port A insertion and adverse events of heme arginate infusion are shown as red triangle with given examined date, Port A-related infection is shown as a green oval. Serum ferritin is indicated as yellow square. Transferrin saturation index (% of serum iron relative to TIBC) is shown as blue triangle. Kidney function is recorded as eGFR level, and sizes of left (L) and right (R) side of kidney along with examined date. Liver function is recorded as ALT and AST levels, and parenchymal liver disease score along with examined date.

Time course descriptions of individual cases are illustrated in Figures 1, 2; Supplementary Figures S1–S3). To illustrate the safety and efficacy of HA prophylaxis, two cases (#3 and #5) are described in detail below (Figures 1, 2).

(*Case #3*) A 21–25-year-old female with disease onset in 2011 at age 16 years, initially presented with persistent sharp pain at the sacral region accompanied by postprandial vomiting and epigastric pain radiating to the back. Hepatic porphyria was suspected due to her AIP family history (daughter of patient 1), and AIP was confirmed after measuring relevant enzyme activity and undergoing genetic testing 3 weeks later. After experiencing 17 recurrent attacks requiring 30 doses of HA treatment within 1 year between 2013 and 2014, weekly prophylactic HA was initiated in January 29, 2014 (Figure 1).

(*Case #5*) A 51–55-year-old female was first admitted to CGMH in 2005 for intermittent lower abdominal pain, lethargy, general malaise and pricking pain for 1 month. She was first diagnosed with AIP at age 38 and has been receiving HA treatment for porphyric attacks since. Her first severe acute attack occurred in 2005 and she was hospitalized for 14 doses of HA treatment (Figure 2). Frequent attacks persisted during menopause (2015), and AARs were similar before and during the first year of menopause (6.04 and 6.46, respectively) (Supplementary Table S2). In 2017, her AAR was 10.06 and both the frequency (10 attacks, 19 HA doses in total) and duration of each attack increased, requiring HA for consecutive days. The patient was hospitalized for 8 days in November 2017. The treating physician recommended prophylactic HA treatment and regular weekly infusions began in December 2017, lasting until now (Figure 2).

### Prophylactic Treatment

(*Case #3*) HA prophylaxis reduced AAR in this patient from 17.06 before prophylaxis to 1.61 afterwards, and annual HA doses decreased from 25.25 to 2.07 doses/year (Table 2). Her kidney function by eGFR was good at disease onset and remained relatively normal over 4.36 years HA prophylactic course (Figure 1; Supplementary Table S3). Aminotransferase levels were higher than the upper limit at disease onset in 2011, but returned to normal from 2012 to date (Figure 1; Supplementary Table S3). Her first port-A was inserted in 2011 for HA treatment, and was replaced twice in 2015 and 2017 due to infections (Figure 1). On June 13, 2018, this patient was recruited to participate in a Phase 3 clinical trial (ENVISION trial of givosiran), and consequently discontinued HA prophylaxis (see discontinuance protocol below).

(*Case #5*) Prophylactic HA treatment effectively prevented porphyric attacks in this patient. No episodes of porphyric attacks occurred after initiation of HA prophylaxis in 2018. AAR was reduced from 10.06 to 0.00, and was sustained throughout 2.58 years of follow-up (Table 2). Renal and kidney function remained stable after HA prophylaxis (Supplementary Tables S3, S4). She received Port-A-cath insertion for HA infusions in 2012 and one Port A replacement was done in 2018. There was an incidence of secondary iron overload, however no signs of end-organ effects were observed after clinical investigation. No other adverse events or infection occurred during the follow-up period (Figure 2).

### Prophylaxis Discontinuation

(*Case #3*) Upon discontinuing HA prophylaxis to participate in a Phase 3 clinical trial for givosiran, this patient was subjected to a 4-week washout period and randomized double-blinded treatment (placebo) for 24 weeks according to the trial protocol (Balwani et al., 2020). During this time, the patient had 18 attacks requiring 30 HA doses (Figure 1). Per trial protocol, an open-label treatment period succeeded the randomized treatment phase, and the patient received givosiran from Jan 16, 2019 to her most recent mid-2020 visit (Figure 1). The clinical results of the trial open-label period are summarized in Figure 1, Supplementary Table S5. In short, 13 porphyric attacks occurred during the open-label period, and only 13 doses of HA were required.

## Discussion

Acute porphyric attacks of AIP are devastating, disruptive, and can lead to long-term complications that reduce patients’ quality of life. In the present study, prophylactic HA infusions given regularly and managed properly provided substantial and long-term clinical benefit to carefully-selected patients. Both the frequency and the severity of porphyric attacks were reduced by prophylactic HA infusions. The safety of HA prophylaxis was demonstrated through the absence of complications and the stability of organ function throughout long-term treatment. Common complications during the course of treatment were venous access issues (infection, thrombosis) that were managed easily. Patients’ liver and kidney function remained stable throughout 2.6–14.6 years of prophylactic HA therapy and no patients developed end-stage renal disease, liver failure or hepatocellular carcinoma. In two patients (cases 1 and 2; Supplementary Figures S3, S4), the frequency and severity of porphyric attacks by prophylactic HA was further reduced during and after menopause, showing that prophylactic HA therapy is effective in both pre- and postmenopausal women.

An audit of prophylactic heme use in England reported that patients had a median of 12 acute attacks requiring hospital admission prior to initiation of heme prophylaxis (Marsden et al., 2015). However, the candidate selection criteria for prophylactic hematin or HA therapy vary between 3-6 attacks per year in previous studies and are determined by clinician discretion. A 40-year retrospective review of a medical records database in the Netherlands indicated that prophylactic heme therapy was initiated in recurrent patients having more than 4 porphyric attacks per year (Neeleman et al., 2018). A recent prospective case series enrolled patients who had at least 3 acute porphyric attacks within the year prior to prophylactic heme treatment (Yarra et al., 2019). A review advised physicians to consider prophylactic heme therapy for recurrent patients with more than 4 attacks per year or female patients with menstrual-associated cyclic attacks (Balwani et al., 2017). In the present study, scheduled prophylactic HA doses was considered for female AIP patients with at least 9 acute attacks in the year prior. This was because we wanted to focus on patients with true refractory and menstrual cycle-related acute attacks, and whose frequent admission to hospital have significantly disrupt home-life, and work (more than 9 porphyric attacks in the year prior is roughly one hospitalization every 1.5 months on average). We trust that our candidate survey and selection mechanism helped to facilitate the satisfactory outcomes reported herein.

Results of the present study demonstrated that HA prophylaxis reduced AAR and the need for acute HA therapy by 50–100%. While there has been one study which reported use of heme arginate increased the frequency of recurrent porphyric attacks (Schmitt et al., 2018), effectiveness were reported by other authors. Marsden et al. (2015) observed a range of 0–75 heme doses for acute porphyric attacks in 22 patients with AIP before prophylactic heme treatment, which decreased to 0–20 during HA prophylaxis (Marsden et al., 2015). AIP patients receiving prophylactic heme therapy in a real-world study using the MarketScan claims database had significantly lower AAR and attack duration than those receiving acute HA treatments only (Blaylock et al., 2020). Another case series reported decreases of 75–100% in acute attacks and inpatient admissions during an 11-month weekly prophylactic heme infusion treatment (Yarra et al., 2019). The present study is in support of the view that HA prophylaxis effectively prevent frequent porphyric attacks and reduce attack severity.

Venous access devices represent an area of concern for regular heme infusions. In a small patient cohort undergoing prophylactic heme regimens for porphyria, the median number of venous access devices used per patient was 2 (range: 1–15 devices over 1–150 months) with mean life-span 1.2 years per device (Marsden et al., 2015). A separate study involving 11 AIP patients with recurrent attacks found that 73% required placement of Port-A catheters to secure venous access, but this was not an absolute limitation for heme therapy (Neeleman et al., 2018). The multinational study EXPLORE demonstrated that of 50% of patients received prophylactic heme, 40% received central venous catheter placement (Gouya et al., 2020); of these, 3 and 4% experienced infectious and thrombotic events, respectively, associated with the central venous catheter. In the present study, Port-A Catheters were placed in all 5 patients, and related infection and thrombosis involved port-A catheter replacement. One patient received 11 port-A catheters over a course of 8 years (Supplementary Figure S4), showing that while venous access-related complications do occur, they are manageable and tolerable.

Puberty, the luteal phase, and hormone replacement are well-recognized precipitating factors for acute porphyric attacks (Andersson et al., 2003; Balwani et al., 2017). and affected patients may either recover spontaneously or enter remission during menopause. A population-based study investigating AIP in women in Sweden found that 50% of women reported reduced symptoms after menopause (Andersson et al., 2003; Balwani et al., 2017). In another Swedish population-based study, 7% of women still experienced post-menopausal attacks (Bylesjo et al., 2009). These findings resonate with the effectiveness of gonadorelin (GnRH) prophylaxis in preventing AIP, for which an audit reported that in 50% of patients (11/22) who considered the treatment ineffective and intolerable, the main complaint was estrogen deficiency (Schulenburg-Brand et al., 2017). In contrast, AAR decreased in almost all patients (19/22) in a UK audit of prophylactic heme therapy, and three patients were being weaned off the drug during remission (Marsden et al., 2015). While 68% (15/22) of patients who received prophylactic heme in the UK audit had tried GnRH previously but discontinued it due to ineffectiveness and adverse events, only one patient (4.5%) in the study of Schulenburg-Brand et al. (2017) found the prophylactic hemin regimen unsatisfactory (Schulenburg-Brand et al., 2017). In the present study, case #5 continued to experience frequent attacks after menopause, and was later treated successfully with prophylactic HA; notably, two other patients (cases 1 and 2) who continued prophylactic HA treatment after menopause experienced reduced attack frequency and severity, suggesting that prophylactic HA may have greater clinical potential in preventing porphyric attacks than GnRH therapy.

The recently approved ALAS-1 RNAi strategy has shown promise, reducing up to 74% of acute attacks in a phase III placebo-controlled trial (Balwani et al., 2020). The adverse events associated with ALAS-1 RNAi include elevation of serum aminotransferase levels, changes in serum creatinine and eGFR suggesting kidney function issues, and injection-site reactions (Balwani et al., 2020). More recently, there have been rare instances concerning severe adverse events including pancreatitis, and homocysteinemia in givosiran-treated porphyria patients (Ventura et al., 2020). A recent study by To-Figueras et al. (2021) showed that while dysregulation of homocysteine homeostasis was also observed in AIP patients receiving heme arginate, givosiran induced an aggravation of the dysregulation. Incidences of secondary iron overload occurred during prophylactic HA treatment in our patients, however, there were only subjective complaints of skin pigmentation from young AIP patients, and no other remarkable end-organ effects of secondary iron overload were noted. In the present study, comparable reductions in AAR were observed in a single patient (case #3) who participated in a clinical trial of givosiran and no remarkable changes were noted in liver and kidney function, no severe adverse event was reported during prophylactic HA treatment nor givosiran treatment period.

The financial burden of treatment for patients with AIP has been the focus of several recent studies (Gouya et al., 2018; Blaylock et al., 2020; Massachi et al., 2020). While FDA-approved givosiran has been shown to reduce the severity and frequency of porphyric attacks in acute hepatic porphyria (Balwani et al., 2020), hemin was found to be the less costly option compared to givosiran for AIP patients due accessibility and current cost for use (Massachi et al., 2020). Based on the published study by Massachi et al., 2020, the total cost of care with hemin for patients with single/multiple attacks per year, and hemin prophylaxis was between 46 and 92% lower compared to givosiran treatment in (Massachi et al., 2020). The information provided in their study may help inform economic decision making. Note however, the cost of complications, side effects, adverse events for either treatments were not accounted for. In addition, the indirect burden on time and travel required to receive weekly HA prophylaxis were also not considered.

This study has a few limitations, including its retrospective nature and small sample size. Not all patients were followed to post-menopausal status and one patient experienced discontinuance of the prophylactic HA infusions. We acknowledge the potential increase in the elimination half-life of heme arginate after repeated infusion, and for this reason weekly prophylactic HA infusions is not a current standard protocol for the frequent and repetitive menstrual cycle-associated porphyric attacks in AIP patients. Prospective evaluation of a larger number of patients with AIP receiving prophylactic HA therapy is needed to confirm results of the present study and add further insight into the potential for preventing porphyric attacks in pre- and post-menopausal women.

## Conclusion

Regular weekly HA infusions demonstrate long-term clinical benefits in reducing the severity and frequency of porphyric attacks in women with AIP. Patients had fewer episodes that required healthcare visits or acute HA treatment and had stable renal and liver function during the follow up period. HA prophylaxis provides a safe and effective strategy for managing patients with AIP.

## Data Availability

The original contributions presented in the study are included in the article/Supplementary Material, further inquiries can be directed to the corresponding author.
