# Supplementary material for: Prophylactic Heme Arginate Infusion for Acute Intermittent Porphyria
Source: Front Pharmacol. 2021 Oct 6;12:712305. doi: 10.3389/fphar.2021.712305 (PMC8526969; doi:10.3389/fphar.2021.712305)
Supplement: Supplementary file 1 [file DataSheet1.docx]

**Supplemental Figures**

**Figure S1. The tracked information of AIP severity, record of Port A insertion, serum ferritin, transferrin saturation index (% of serum iron relative to TIBC) and adverse events of heme arginate infusion, monitoring of kidney function and liver function for patient #1.**

**Figure S2. The tracked information of AIP severity, record of Port A insertion, serum ferritin, transferrin saturation index (% of serum iron relative to TIBC) and adverse events of heme arginate infusion, monitoring of kidney function and liver function for patient #2.**

**Figure S3. The tracked information of AIP severity, record of Port A insertion, serum ferritin, transferrin saturation index (% of serum iron relative to TIBC) and adverse events of heme arginate infusion, monitoring of kidney function and liver function for patient #4.**

AIP severity is identified by numbers of attacks and total heme arginate doses for porphyric attacks treated. Red dots indicate AIP severity status within 1-year before receiving prophylactic heme arginate. A dotted frame marks the initiation date of initial prophylactic heme arginate. Port A insertion and adverse events of heme arginate infusion are shown as a red triangle with given examined date, thrombophlebitis as a purple cylinder, and Port A infection as a green oval. Serum ferritin is indicated as yellow square. Transferrin saturation index (% of serum iron relative to TIBC) is shown as blue triangle. Kidney function is recorded as eGFR level, and sizes of left (L) and right (R) side of kidney along with examined date. Liver function is recorded as ALT and AST levels, and parenchymal liver disease score along with examined date.

**Supplementary Tables**

**Table S1. 24-hr urine test of PBG and ALA for Case #1 to #5.**

**Table S2. AIP severity for the time period before and after initiating HA prophylactic treatment.**

**Table S3. Changes in kidney function before and after heme arginate prophylactic treatment.**

**Table S4. Change in liver function before and after heme arginate prophylactic treatment.**

**Table S5. Case #3: Summary of changes in AAR†, severity of attack (number of attacks and doses used) before and after heme arginate prophylaxis and givosiran prophylactic treatment**

**Figure S1. The tracked information of AIP severity, record of Port A insertion****, serum ferritin, transferrin saturation index (% of serum iron relative to TIBC) and adverse events of heme arginate infusion, monitoring of kidney function and liver function for patient #1.**


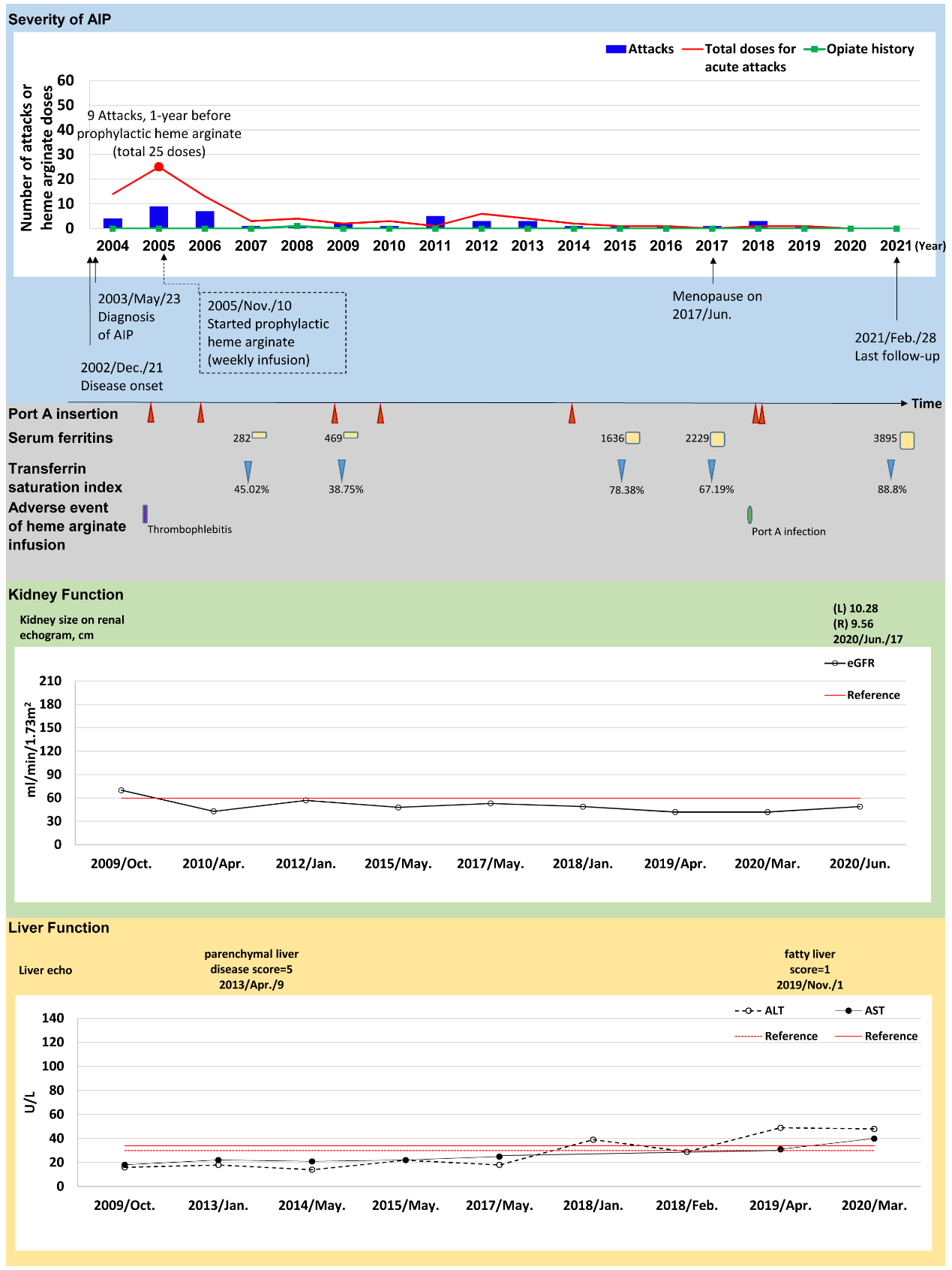


**Figure S2. The tracked information of AIP severity, record of Port A insertion, serum ferritin, transferrin saturation index (% of serum iron relative to TIBC) and adverse events of heme arginate infusion, monitoring of kidney function and liver function for patient #2.**


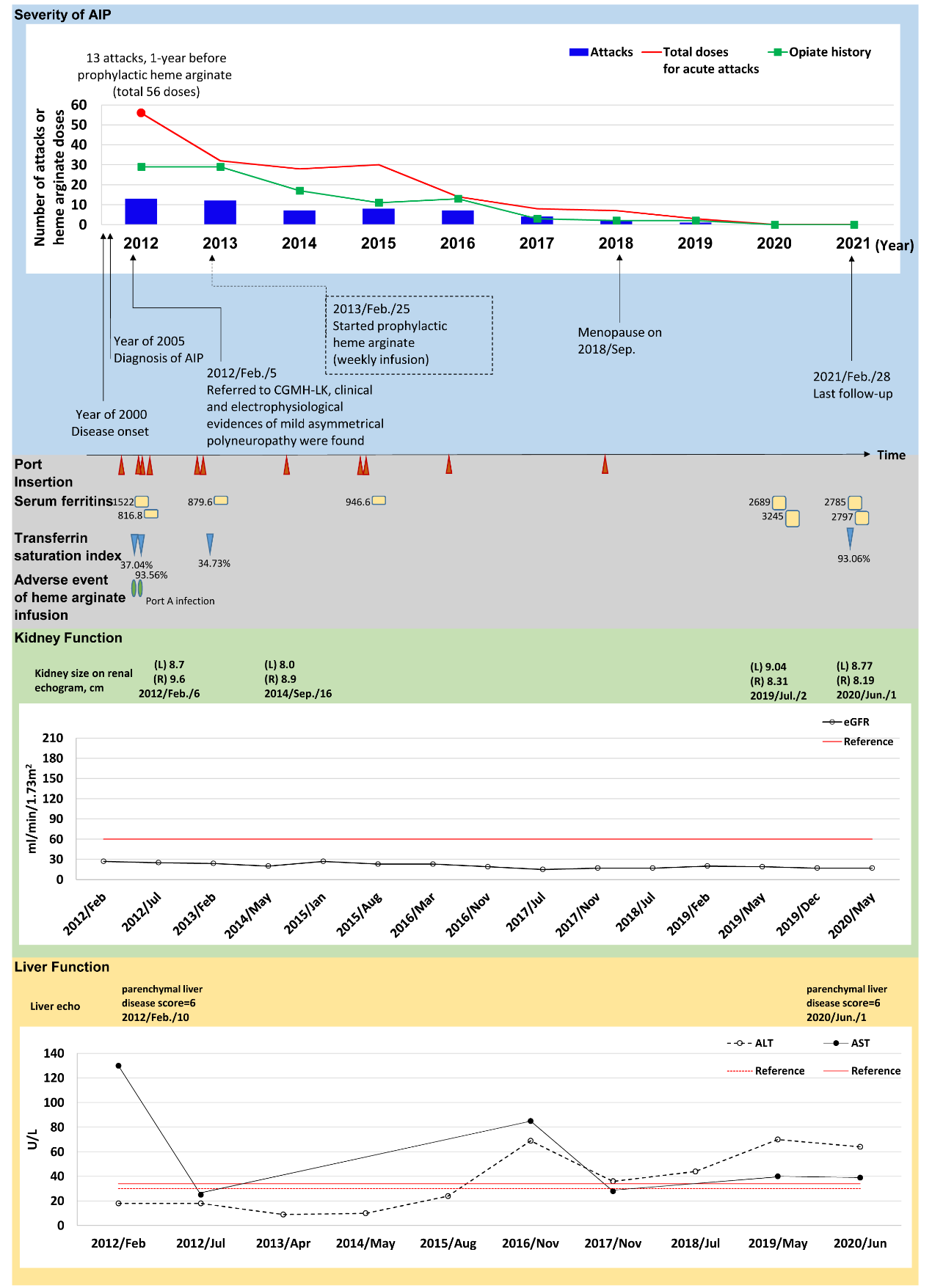


**Figure S3. The tracked information of AIP severity, record of Port A insertion, serum ferritin, transferrin saturation index (% of serum iron relative to TIBC) and adverse events of heme arginate infusion, monitoring of kidney function and liver function for patient #4.**

**
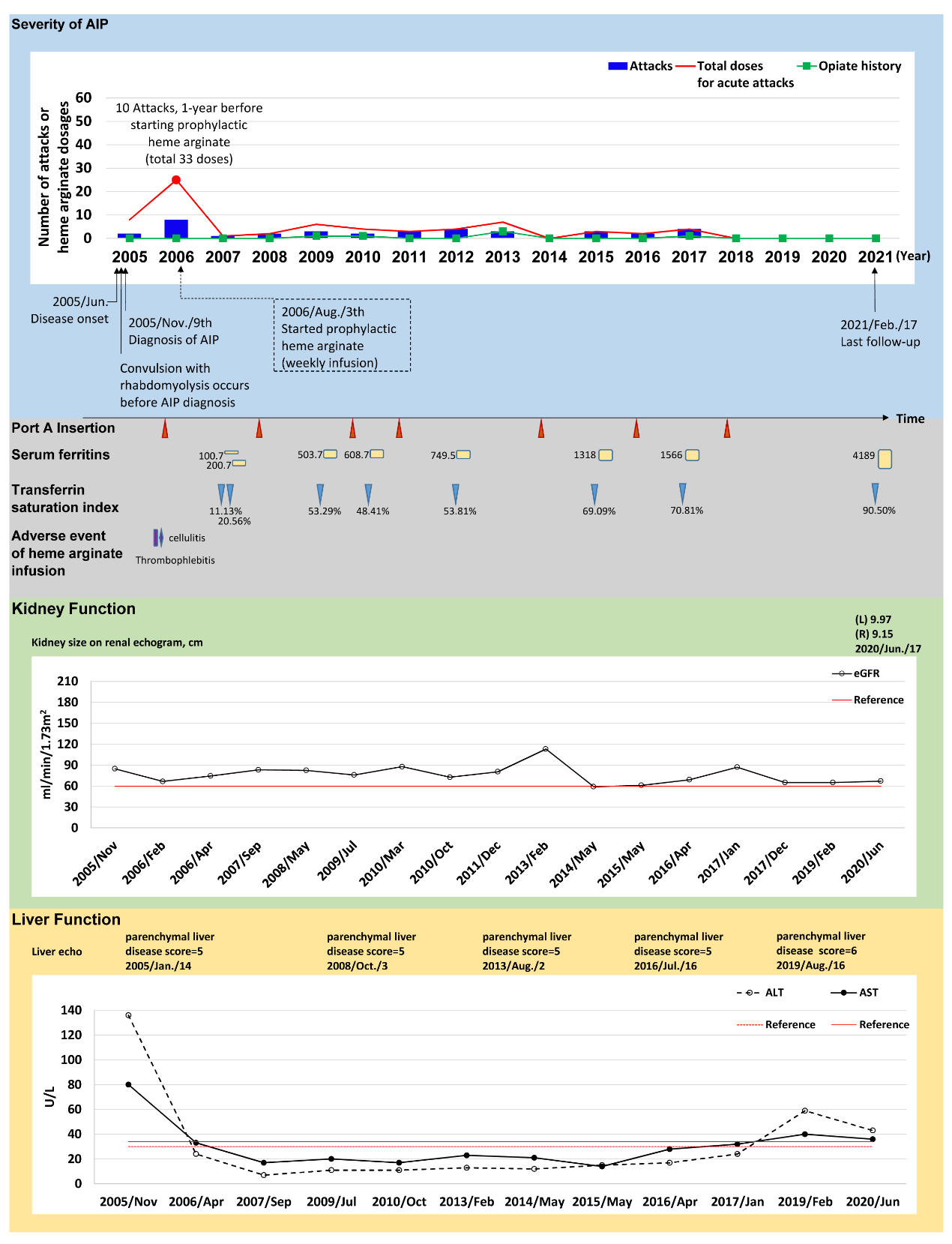
**

**Supplementary Table S1**. 24-hour urine test of PBG and ALA for Case #1 to #5

|  | **Case # 1** | | | | | | | | | | | | | | | | | | | | | | | | | | |
| --- | --- | --- | --- | --- | --- | --- | --- | --- | --- | --- | --- | --- | --- | --- | --- | --- | --- | --- | --- | --- | --- | --- | --- | --- | --- | --- | --- |
| Date | May23, 2003 | Dec.5, 2003 | | Jan.17, 2004 | | May28, 2004 | Sep.4, 2004 | | Oct.29, 2004 | | Jan.5,  2005 | | Feb.18, 2005 | | Mar.23, 2005 | | Apr.21, 2005 | | May23, 2005 | | Jul.18,  2005 | | Jul.22, 2005 | | *Nov.10, 2005 | | Dec.11, 2006 |
| PBG | 113 | 147.5 | | 34.96 | | 78.9 | 154.7 | | 133.5 | | 71.1 | | 97.7 | | 90.2 | | 144.9 | | N/A | | 129.2 | | 34.7 | | N/A | | 176.9 |
| δ-ALA | 58.2 | 70.6 | | 34.06 | | 34.7 | 98.5 | | 81.97 | | 30.9 | | 26.6 | | 32 | | 81.2 | | 20.9 | | 70.1 | | 11.54 | | N/A | | 45.1 |
| Date | May18 2007 | May25, 2007 | | Jun.1, 2007 | | Jun.8, 2007 | Jun.15, 2007 | | Jun.22, 2007 | | Jul.6,  2007 | | Jul.9,  2007 | | Jul.16,  2007 | | Jul.24,  2007 | | Aug.1, 2007 | | Aug.9,  2007 | | Aug.24, 2007 | | Feb.15, 2008 | | Jan.26, 2018 |
| PBG | 68.6 | 77.6 | | 107.1 | | 72.2 | 87.9 | | 92.3 | | 81.3 | | 38.8 | | 33.89 | | 85.52 | | 72.97 | | 58.2 | | 76.4 | | 49.65 | | 93.82 |
| δ-ALA | 43 | 31.4 | | 39.8 | | 22.8 | 30.3 | | 32 | | 47.1 | | 12.6 | | 12.46 | | 27.75 | | 23.32 | | 18.4 | | 31.4 | | 25.23 | | 20.62 |
| Date | Apr.26, 2019 | | Feb.19, 2021 | |  |  | |  | |  | |  | |  | |  |  |  | |  | |  | |  | |  | |
| PBG | 20 | | 15.51 | |  |  | |  | |  | |  | |  | |  |  |  | |  | |  | |  | |  | |
| δ-ALA | 5 | | 7.2 | |  |  | |  | |  | |  | |  | |  |  |  | |  | |  | |  | |  | |
|  | **Case # 2** | | | | | | | | | | | | | | | | | | | | | | | | | | |
| Date | Feb.6, 2012 | | Feb.26, 2012 | | Mar.20, 2012 | Apr.3, 2012 | | Apr.16, 2012 | | May9, 2012 | | Jun.1,  2012 | | Jul.3,  2012 | | Aug.10, 2012 | Oct.30, 2012 | Nov.25, 2012 | | Dec.19, 2012 | | Jan.1,  2013 | | Jan.25, 2013 | | Feb.20, 2013 | |
| PBG | 134.99 | | 60.47 | | 72.69 | 32.25 | | 84.23 | | 67 | | 64.28 | | 52.7 | | 24.59 | 46.38 | 54.59 | | 76.8 | | 46.04 | | 9.55 | | 19.59 | |
| δ-ALA | 34.76 | | 5.27 | | 13.27 | 7.02 | | 7.37 | | 8.63 | | 7.63 | | 4 | | 3.57 | 5.87 | 8.69 | | 15.48 | | 4.19 | | 1.16 | | 2.3 | |
| Date | *Feb.25, 2013 | Mar.11, 2013 | | Apr.30, 2013 | | May10, 2013 | Jun.14, 2013 | | Jul.9,  2013 | | Aug.2,  2013 | | Aug.29, 2013 | | Oct.9,  2013 | | Nov.14, 2013 | | Feb.10, 2014 | | May8,  2014 | | Jun.8, 2014 | | Jul.24, 2014 | | Aug.20, 2014 |
| PBG | N/A | 90.75 | | 44.67 | | 29 | 22.34 | | 36.03 | | 17.89 | | 36.06 | | 63.47 | | 29.13 | | 26.25 | | 31.56 | | 77.31 | | 13.6 | | 55.77 |
| δ-ALA | N/A | 12.27 | | 6.21 | | 7.89 | 2.91 | | 2.97 | | 2.64 | | 3.64 | | 7.18 | | 3.51 | | 3.27 | | 2.86 | | 6.96 | | 3.33 | | 6.81 |
| Date | Oct.15, 2014 | Jan.8, 2015 | | Feb.27, 2015 | | May16, 2015 | Aug.24, 2015 | | Sep.26, 2015 | | Nov.20, 2015 | | Feb.3, 2016 | | Jul.16,  2016 | | Nov.19, 2016 | | Jul.21, 2017 | | Nov.15, 2017 | | Jan.10, 2018 | | Jul.18, 2018 | | May13, 2019 |
| PBG | 46.53 | 20.24 | | 55.85 | | 101.64 | 105.66 | | 50.52 | | 23.67 | | 58.38 | | 14.75 | | 22.02 | | 15.56 | | 69.43 | | 40.24 | | 44.53 | | 70.72 |
| δ-ALA | 5.44 | 3.87 | | 12.42 | | 14.78 | 26.85 | | 10.81 | | 3 | | 6.52 | | 1.76 | | 4.03 | | 2.24 | | 10.35 | | 4.24 | | 4.86 | | 8.17 |
|  | **Case #3** | | | | | | | | | | | | | | | | | | | | | | | | | | |
| Date | Nov.25, 2011 | Dec.6, 2011 | | Jan.6, 2012 | | Nov.23, 2012 | Dec.11, 2012 | | Jan.17, 2013 | | Dec.6,  2013 | | Jan.24, 2014 | | *Jan.29,  2014 | | May13, 2018 | | Feb.14, 2021 | |  | |  | |  | |  |
| PBG | 101.73 | 154.26 | | 160.11 | | 125 | 143.75 | | 125.8 | | 75.45 | | 39.67 | | 26.1 | | 122.89 | | 14.83 | |  | |  | |  | |  |
| δ-ALA | 33.1 | | 55.73 | | 65.73 | 49.31 | | 79.8 | | 39.31 | | 11.66 | | 8.52 | | 7.67 | 41.79 | 5.82 | |  | |  | |  | |  | |
|  | **Case # 4** | | | | | | | | | | | | | | | | | | | | | | | | | | |
| Date | Oct.13, 2005 | | Nov.14, 2005 | | Nov.21, 2005 | Jan.2,  2006 | | Feb.2, 2006 | | Apr.3, 2006 | | May1, 2006 | | May30, 2006 | | Jun.26, 2006 | Jul.22, 2006 | *Aug.3, 2006 | | May14, 2007 | | May21, 2007 | | May28, 2007 | | Jun.4,  2007 | |
| PBG | 77.2 | | 72.6 | | 18.75 | 62.2 | | 100.2 | | 16.2 | | 22.3 | | 16 | | 67.4 | 52.4 | N/A | | 72.1 | | 47.9 | | 56.2 | | 31.8 | |
| δ-ALA | N/A | | 20.6 | | 3.76 | 14.8 | | 30.2 | | 6.4 | | 9.18 | | 7.1 | | 21.2 | 10.3 | N/A | | 19.9 | | 16.6 | | 18.1 | | 11 | |
| Date | Jun.11, 2007 | | Jun.18, 2007 | | Jun.25, 2007 | Jul.2,  2007 | | Sep.6, 2007 | | Sep.7, 2007 | | Sep.8, 2007 | | Sep.10, 2007 | | Sep.11, 2007 | Sep.12, 2007 | May15, 2008 | | Oct.25, 2008 | | Jul.30, 2009 | | Mar.7, 2010 | | Feb.15, 2013 | |
| PBG | 56.5 | | 56.1 | | 47.6 | 38.5 | | 26.8 | | 24.37 | | 35.59 | | 67.85 | | 52.06 | 37.22 | 76.57 | | 33.1 | | 51.08 | | 25.4 | | 69.38 | |
| δ-ALA | 16.4 | | 12.2 | | 12 | 15.8 | | 7.6 | | 9.3 | | 11.39 | | 18.71 | | 13.01 | 5.22 | 31.52 | | 8.48 | | 18.6 | | 6.16 | | 17.42 | |
| Date | Nov.18, 2013 | | Nov.25, 2013 | | Dec.2, 2013 | Dec.9, 2013 | | Dec.16, 2013 | | Dec.23, 2013 | | Jan.7,  2014 | | Jan.13, 2014 | | Jan.20, 2014 | Feb.5, 2014 | Dec.14, 2017 | | Apr.29, 2019 | | Feb.17, 2021 | |  | |  | |
| PBG | 36.39 | | 51.95 | | 24.58 | 33.06 | | 37.98 | | 60.33 | | 66.1 | | 33.44 | | 56.43 | 35.54 | 85.08 | | 46.69 | | 105.34 | |  | |  | |
| δ-ALA | 8.68 | | 9.26 | | 6.52 | 8.52 | | 7.5 | | 15.65 | | 5.28 | | 6.79 | | 11.67 | 5.76 | 28.48 | | 8.79 | | 47.46 | |  | |  | |
|  | **Case # 5** | | | | | | | | | | | | | | | | | | | | | | | | | | |
| Date | Jun.27, 2005 | Jul.7, 2005 | | Jul.14, 2005 | | Aug.26, 2005 | Mar.23, 2012 | | Sep.24, 2014 | | Feb.16, 2015 | | Mar.23, 2015 | | Aug.15,  2017 | | Nov.2,  2017 | | *Nov.29, 2017 | | Jan.10,  2018 | | Jul.4, 2018 | | Oct.17, 2018 | | May3, 2019 |
| PBG | 52.1 | 27.1 | | 19.1 | | 38.4 | 52.16 | | 101.52 | | 20.88 | | 25.83 | | 22.07 | | 63.49 | | 103.9 | | 24.19 | | 36.04 | | 61.05 | | 35.1 |
| δ-ALA | 63.9 | | 14.9 | | 8.23 | 11 | | 17.65 | | 22.18 | | 3.67 | | 3.82 | | <0.3 | 20.77 | 22.61 | | 4.15 | | 5.18 | | 11.73 | | 8.04 | |

Date, Month-day-year.

Color indicates treatment stage when measurements were collected: Red is at porphyric attack and initiation of heme arginate infusion; Yellow, is during continual heme arginate infusion for acute attack; Green is close to symptom relief or free of attack.

* Heme arginate prophylactic treatment start.

Abbreviations: PBG, Porphobilinogen; δ-ALA, δ-Aminolevulinic acid

Reference for PBG/24-hour urine collection: 0.0 to 2.7 mg/24hrs.

Reference for δ-ALA/24-hour urine collection: 1.5 to 7.5 mg/24hrs.

**Table S2 Summary of AIP severity by case.**

|  | **Attacks** | **Doses for  porphyric attacks** | **Doses/Attacks** | **Duration***  **(years)** | **AAR†** |
| --- | --- | --- | --- | --- | --- |
| **Case #1** |  |  |  |  |  |
| Heme arginate for porphyric attack 1-year before prophylactic treatment | 9 | 25 | 2.78 | 1.00 | 9.**03** |
| Initial heme arginate prophylactic treatment to menopause | 26 | 40 | 1.54 | 11.64 | 2.23 |
| Heme arginate prophylactic treatment from menopause to present | 5 | 2 | 0.40 | 3.00 | 1.67 |
| **Case #2** |  |  |  |  |  |
| Heme arginate for porphyric attack 1-year before prophylactic treatment | 13 | 56 | 4.31 | 1.00 | 13.01 |
| Initial; heme arginate prophylactic treatment until menopause | 40 | 119 | 2.98 | 5.59 | 7.15 |
| Heme arginate prophylactic treatment from menopause to present | 1 | 3 | 3.00 | 1.75 | 0.57 |
| **Case #3** |  |  |  |  |  |
| Heme arginate for porphyric attack 1-year before prophylactic treatment | 17 | 30 | 1.76 | 1.00 | 17.06 |
| Initial heme arginate prophylactic treatment until screening for clinical trial | 7 | 9 | 1.29 | 4.36 | 1.61 |
| Joined clinical trial and stopped heme arginate prophylactic treatment (wash-out 4 weeks and placebo 24 weeks) | 18 | 30 | 1.67 | 0.59 | 30.44 |
| Givosiran (2.5 mg/kg subcutaneous injection monthly) prophylactic treatment | 13 | 13 | 1.00 | 1.45 | 8.94 |
| **Case #4** |  |  |  |  |  |
| Heme arginate for porphyric attack 1-year before prophylactic treatment | 10 | 33 | 3.30 | 1.00 | 10.03 |
| Heme arginate prophylactic treatment | 27 | 36 | 1.33 | 13.91 | 1.94 |
| **Case #5** |  |  |  |  |  |
| Heme arginate for porphyric attacks from diagnosis to menopause | 63 | 89 | 1.41 | 10.43 | 6.04 |
| Heme arginate for porphyric attacks 1 year after menopause | 7 | 7 | 1.00 | 1.08 | 6.46 |
| Heme arginate for porphyric attack 1-year before prophylactic treatment | 10 | 19 | 1.90 | 1.00 | 10.03 |
| After heme arginate prophylactic treatment | 0 | 0 | - | 2.58 | 0.00 |

*Duration, total number of days receiving treatment/365.25.

†AAR, annualized attack rate (=total number of porphyric attacks/duration)

**Table S3. Summary of changes in kidney function before and after heme arginate prophylactic treatment.**

|  | Heme arginate for porphyric attack 1-year before prophylactic treatment | |  | Start heme arginate prophylactic treatment until 2020/Jun./30 | |
| --- | --- | --- | --- | --- | --- |
|  | eGFR, ml/min/1.73m^2^ | Kidney size^*^, cm (L't/R't) |  | eGFR, ml/min/1.73m^2^ | Kidney size^*^, cm (L't/R't) |
| #1 | n/a | n/a |  | 49 | 10.28/9.56 |
| #2 | 24 | 8.7/9.6 |  | 17 | 8.77/8.19 |
| #3 | 144 | n/a |  | 112^a^ | 11.46/11.01^a^ |
| #4 | 74.4 | n/a |  | 67 | 9.97/9.15 |
| #5 | 16 | 7.5/7.8 |  | 12 | 7.8/7.7 |
| ***Average*** | ***64.6*** | ***8.1/8.7*** |  | ***51.4*** | ***9.65/9.12*** |

n/a, not available, eGFR, estimated Glomerular filtration rate (ref. >60 ml/min/1.73m^2^)

^*^Left (L't ) and right (R't) kidney size.

^a^ For Case #3, duration was set as date of starting heme arginate prophylactic treatment until date of screening for clinical trial (2018/Jun./8).

**Table S4 Summary of changes in liver function before and after heme arginate prophylactic treatment.**

|  | Heme arginate for porphyric attack 1-year before prophylactic treatment | | |  | Initial heme arginate prophylactic treatment until menopause | | |  | Heme arginate prophylactic treatment from menopause to 2020/Jun./30 | | |  | Initial heme arginate prophylactic treatment until 2020/Jun./30 | | |
| --- | --- | --- | --- | --- | --- | --- | --- | --- | --- | --- | --- | --- | --- | --- | --- |
|  | ALT, U/L | AST, U/L | parenchymal liver disease score |  | ALT, U/L | AST, U/L | parenchymal liver disease score |  | ALT, U/L | AST, U/L | parenchymal liver disease score |  | ALT, U/L | AST, U/L | parenchymal liver disease score |
| #1 | n/a | n/a | n/a |  | 18 | 25 | 5 |  | 48 | 40 | 1^*^ |  | 48 | 40 | 1^*^ |
| #2 | 18 | 25 | 6 |  | 44 | 28 | n/a |  | 64 | 39 | 6 |  | 64 | 39 | 6 |
| #3 | 20 | 20 | n/a |  | - | - | - |  | - | - | - |  | 19^b^ | 16^b^ | 5^b^ |
| #4 | 24 | 33 | 5 |  | - | - | - |  | - | - | - |  | 43 | 36 | 6 |
| #5 | 27 | 28 | 5 |  | - | - | - |  | 13 ^a^ | 22^a^ | 5^a^ |  | 10 | 21 | 5 |
| ***Average*** | ***22.3*** | ***26.5*** | ***5.3*** |  | ***31.0*** | ***26.5*** | ***5.0*** |  | ***41.7*** | ***33.7*** | ***5.5*** ^c^ |  | ***36.8*** | ***30.4*** | ***5.5*** |

n/a, not available, ALT, Aspartate aminotransferase (ref. 0-34 U/L), AST, Alanine aminotransferase Ref, 0-30 U/L), ^*^fatty liver score.

^a^ For case #5, data of heme arginate treatment for porphyric attacks were not included.

^b^ For Case #3, duration was set as date of starting heme arginate prophylactic treatment until date of screening for clinical trial (2018/Jun./8)

^c^ Only parenchymal liver disease was considered.

**Table S5. Case #3: Summary of changes in AAR†, severity of attack (number of attacks and doses used) before and after heme arginate prophylaxis and givosiran prophylactic treatment**

| **Time Period** | **Duration***  **(year)** | **Doses/Attack**  **(n)** | **Total HA dose for the period (n)** | **AAR** |
| --- | --- | --- | --- | --- |
| Before HA prophylaxis | 1.59 | 1.71 | 37.74 | 22 |
| 1-year before HA prophylaxis | 1.00 | 1.76 | 30.09 | 17.06 |
| HA prophylactic period | 4.36 | 1.29 | 2.06 | 1.61 |
| Givosiran prophylactic period | 1.45 | 1.00 | 8.97 | 8.94 |

*Duration, total number of days receiving treatment/365.25.

†AAR, annualized attack rate = total number of porphyria attacks/year for each time period)
